# Automated cephalometric landmark detection with confidence regions using Bayesian convolutional neural networks

**DOI:** 10.1186/s12903-020-01256-7

**Published:** 2020-10-07

**Authors:** Jeong-Hoon Lee, Hee-Jin Yu, Min-ji Kim, Jin-Woo Kim, Jongeun Choi

**Affiliations:** 1 School of Mechanical Engineering, Yonsei University, 50 Yonsei Ro, Seodaemun Gu, Seoul, 03722 Republic of Korea; 2 Department of Orthodontics, School of Medicine, Ewha Womans University, Anyangcheon-ro 1071, Yangcheon-gu, Seoul, 07985 Republic of Korea; 3 Department of Oral and Maxillofacial Surgery, School of Medicine, Ewha Womans University, Anyangcheon-ro 1071, Yangcheon-gu, Seoul, 07985 Republic of Korea

**Keywords:** Artificial neural networks, Bayesian method, Cephalometry, Orthodontics, Machine vision, Deep learning, Artificial intelligence, Orthodontic(s), Radiography, Orthognathic/orthognathic surgery, Oral & maxillofacial surgery, Dental anatomy

## Abstract

**Background:**

Despite the integral role of cephalometric analysis in orthodontics, there have been limitations regarding the reliability, accuracy, etc. of cephalometric landmarks tracing. Attempts on developing automatic plotting systems have continuously been made but they are insufficient for clinical applications due to low reliability of specific landmarks. In this study, we aimed to develop a novel framework for locating cephalometric landmarks with confidence regions using Bayesian Convolutional Neural Networks (BCNN).

**Methods:**

We have trained our model with the dataset from the ISBI 2015 grand challenge in dental X-ray image analysis. The overall algorithm consisted of a region of interest (ROI) extraction of landmarks and landmarks estimation considering uncertainty. Prediction data produced from the Bayesian model has been dealt with post-processing methods with respect to pixel probabilities and uncertainties.

**Results:**

Our framework showed a mean landmark error (LE) of 1.53 ± 1.74 mm and achieved a successful detection rate (SDR) of 82.11, 92.28 and 95.95%, respectively, in the 2, 3, and 4 mm range. Especially, the most erroneous point in preceding studies, Gonion, reduced nearly halves of its error compared to the others. Additionally, our results demonstrated significantly higher performance in identifying anatomical abnormalities. By providing confidence regions (95%) that consider uncertainty, our framework can provide clinical convenience and contribute to making better decisions.

**Conclusion:**

Our framework provides cephalometric landmarks and their confidence regions, which could be used as a computer-aided diagnosis tool and education.

## Background

From the classic to contemporary orthodontics, treatment modalities of analyzing the spatial relationships of teeth, jaws, and cranium rely heavily on the cephalometry. Using standardized cephalometric x-ray, predefined anatomic landmarks are marked so that various orthodontic and facial morphometric analyses can be applied for the diagnosis and treatment planning. Despite the several methodological limitations such as nonlinear magnification and distortion of images, its integral role in orthodontics, as well as orthognathic and facial plastic surgery is indisputable [1–3].

The accuracy of marked cephalometric landmarks can affect the results of the clinical performance of analyses and resulting treatment decisions [4, 5]. Since the boundary of distinguishing abnormalities is concentrated within the unit range of millimeters or several degrees, even a slight error can have the potential to cause misclassification that can lead to malpractice. What makes this even more challenging is that a human skull is a highly sophisticated 3D object, whereas in a lateral cephalogram which has its model projected onto a sagittal plane, causes cubic features of the direction perpendicular to the plane to be overlapped [6, 7].

Although cephalometric tracing is generally conducted by trained orthodontists in clinical practice, numerous reports were concerned about the significant intra- and inter-observer variabilities among them due to the various limitations and its time-consuming nature [7, 8]. To achieve better accuracy and reliability for cephalometric tracing, the need for fully automatic tracing software has constantly been raised [9].

Throughout the decades, there have been several studies on computer-aided landmark detection. Template matching and gray-scale morphological operator was used for works of Cardillo and Sid-Ahmed, et al [10]. Ibragimov et al. used Random Forest and Game Theoretic techniques on the ISBI grand challenge 2014 for good performance [11]. Tree based approaches such as random forest regression with a hierarchic framework and binary pixel classification with the randomized tree were used by Chu et al. and Vandaele et al. respectively [12, 13]. Despite these efforts, the developed methods have shown limitations over the accuracy and the uncertainty issues, proposing around 70% of estimated landmarks within the clinically acceptable range of 2 mm distance from the ground truth points [9].

Through the last decade, various clinical fields have reported an increase in clinical efficiency according to the application of artificial intelligence. In particular, recent studies in the dental field have shown excellent performance in clinical applications as a diagnostic aid system for deep learning models [14, 15]. Many deep learning based computer aided landmark detection studies have performed better than previous studies. Lee et al. and Arik et al. adopted the basic concept of CNN for pixel classifying algorithm [16, 17]. U-shaped deep CNN has widely been used to precisely estimate their points [18–21]. However, in the case of a single CNN model, there is no uncertainty provided over model calculations, which works as a medical obstacle for the users to accept the outcome produced from the algorithm.

In this paper, we propose the novel framework for locating cephalometric landmarks with confidence regions based on uncertainties using Bayesian Convolutional Neural Networks (BCNN) [22]. With Bayesian inference over iterative CNN model calculations, we can derive the confidence region (95%) of an identified landmark considering model uncertainty, and significantly higher the in-region accuracy. Given the uncertainty and confidence areas of the estimated location, clinicians are expected to determine whether the results of the framework are reliable and to make a more accurate diagnosis.

## Methods

### Dataset description

The material used in this study consisted of sets of data from 400 subjects provided by the ISBI 2015 challenge [23] (website: http://www-o.ntust.edu.tw/~cweiwang/ISBI2015/challenge1/). Each set comprised one lateral cephalogram, and two coordinate sets of landmark points manually plotted by two experts respectively (junior and senior orthodontic specialists). The mean intra-observer variability of landmark points was 1.73 and 0.90 mm for two experts [17]. In this study, the mean position of the two points was used as the ground truth, in a way that the inter-observer variability can be compensated. An image had a size of 1935 × 2400 pixels, and one pixel corresponded to a square size of 0.1 mm in length on one side. Each pixel had a single channel gray-scale value range in [0, 255]. To be consistent with the study preceded in the challenge, 150 images proposed from the ISBI were used for training, and the remaining 250 images were tested to examine the overall performance.

This study was performed within the guidelines of the World Medical Association Helsinki Declaration for biomedical research involving human subjects. It was approved by the institutional review board of the Ewha Womans University Medical Center, Seoul, Korea. (EUMC-2017-12-031-002).

### Overall algorithm of the landmark detection framework

**Fig. 1:**
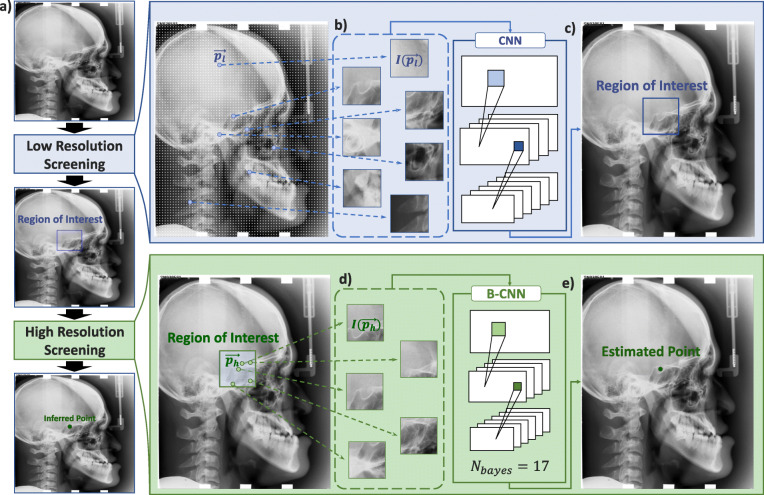
Schematic of the overall detection framework. **a** Original lateral cephalogram (lat ceph) gets downsampled by a factor of 3. **b** From the downsampled lat ceph, image batches ($I\left(\overrightarrow{p_l}\right)$) are sampled with a stride of 3 mm along the width and the height direction from all over the lat ceph. **c** From the LRS calculation, CNN model provides a region of interest for the target landmark to be located in. **d** Every single pixel from the ROI is, again, sampled as an image batch ($I\left(\overrightarrow{p_h}\right)$) to be put into Bayesian CNN(B-CNN) model for iterative calculations. **e** HRS provides the final predicted target position for the target landmark

In this study, the entire framework was divided into two procedures: Low-Resolution Screening (LRS) and High-Resolution Screening (HRS). This allowed for higher performance by separating complex tasks into easier subtasks. The objective of the LRS was to produce the ROI of the corresponding landmark and that of HRS was to estimate the exact landmarks considering the uncertainty. Once the center of the expected region is determined from the LRS, every single pixel within the ROI is then to be judged whether it corresponds to the target landmark point (Fig. 1).

#### Low resolution screening

To produce the ROI of the target landmark from LRS, first, pixel positions were sampled with the stride of 3 mm (30 pixels) along the width and height direction from the entire lateral cephalogram. Since it is not possible to determine whether it is the target landmark or not merely with its pixel value alone, a square image with an *ith* pixel position ($\overrightarrow{p_i}=\left({x}_i,{y}_i\right)$) at the center was constructed (9.1 mm × 9.1 mm) and denoted as $I\left(\overrightarrow{p_i}\right)$. From a CNN model trained for the target landmark k (*C*_*k*_), It is determined whether $I\left(\overrightarrow{p_l}\right)$ resembles the target landmark as the following relation, ${C}_k:I\left(\overrightarrow{p_l}\right)\to \left[0,1\right]$, where the output from the model indicated true or false by corresponding to a value of 1 or 0 respectively.

Let the center point of the ROI of landmark k from LRS be $\left(\hat{x_k^L},\hat{y_k^L}\right)$. Then, for the *ith* position classified as true ($\overrightarrow{p_i^t}$) from LRS among the *n*_*T*_ total true images, it can be determined as the following equation.

$$ \left(\hat{x_k^L},\hat{y_k^L}\right)=\frac{1}{n_T}\sum \limits_{i=1}^{n_T}\overrightarrow{p_i^t} $$

#### High resolution screening – score weighting method

From the center point from LRS $\left(\hat{x_k^L},\hat{y_k^L}\right)$, a square-shaped ROI was constructed based upon a reference side length. The reference side length was set to 40 mm where 99% of the ground truth had positioned within. Every pixel position $\overrightarrow{p_h}$ within the ROI was then sampled and imaged in a similar way to that of LRS, $I\left(\overrightarrow{p_h}\right)$, to be an input for HRS. A Bayesian CNN model of the landmark k, *B*_*k*_, conducted a forward-propagation with the sampled image batches and produced mean (*μ*_*i*_) and the uncertainty (*σ*_*i*_) with respect to the corresponding softmax values of each input. Since those of the pixels which have a higher Bayesian mean and a lower uncertainty are likely to be the target landmark, this study proposed a simple post-processing method – Score Weighting Method – which can successfully consider the relation of the two as the following.

$$ {score}_i=\left({e}^{10{\mu}_i}-1\right)\tanh \left(\frac{\sqrt{\sum_{j=1}^N{\sigma}_j^2}}{s_{\sigma }{\sigma}_i}\right) $$

From the score given to each pixel in the ROI, the final estimated point of the landmark k, $\left(\hat{x_k^H},\hat{y_k^H}\right),$ can be derived. Similar to the mass center, the estimation can be regarded as the ‘score center’ with the equation below.

$$ \left(\hat{x_k^H},\hat{y_k^H}\right)=\frac{\sum_i\left({score}_i\times \overrightarrow{p_{h_i}}\right)}{\sum_i{score}_i} $$

**Fig. 2:**
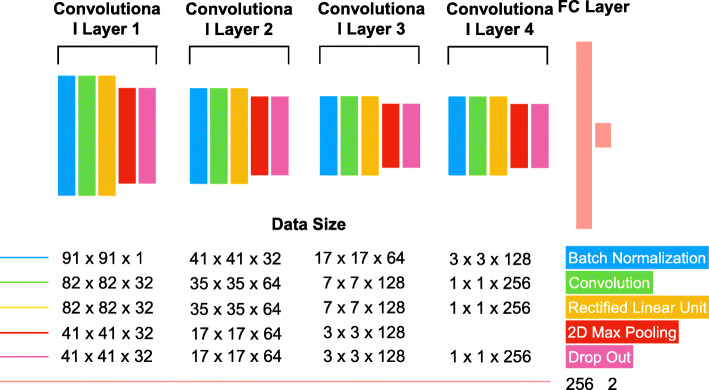
The model architecture of our landmark detecting framework. The architecture has 4 Convolutional Cluster (CC) and 2 Fully Connected (FC) layers. Each CC contains the Batch Normalization layer, Convolution layer, Non-linearity, 2D max-pooling, and dropout in the mentioned order

The model architecture is illustrated in detail in Fig. 2 and the details on the BCNN for estimating uncertainty are provided in the Method Detail of Appendix with the set of formulas.

### Model architecture and training details

BCNN utilizes the geographic characteristics of the input data, similar to that of CNN, which generates information by extracting location characteristics from the kernel at each layer. As shown in the Fig. 2, the entire model has a total of 4 Convolutional Cluster (CC) and 2 Fully Connected (FC) layers. Each CC contains the Batch Normalization layer, Convolution layer, Non-linearity, 2D max-pooling, and dropout in the mentioned order. The Batch normalization layer, the first layer of a CC, normalizes input data batches with respect to their intensity (γ) and bias (β) at the training time. At the test time, it uses trained γ and β to remove the covariance-shifts present in the test data batches. This process results in improving the accuracy of 1 ∼ 2% than without it, and also larger learning rate can be applied so that it takes less time to train [24]. Lastly, the dropout layer randomly restricts the activation of several neurons by the rate the user proposes. Because of its randomness, a key role in Bayesian inference, overfitting can be prevented.

The kernel has a size of 10 at one side each with a depth of 32. As the layer deepens, size reduces from 7, 5, to 1 and the depth increases from 64, 128 to 256 respectively. The last dropout layer of the CC is then connected to the first FC layer. The first FC layer has the same number of neurons with the number of information given from the last dropout layer, and the second FC layer has several neurons identical to the number of model outputs (True, False). The dropout rate, learning rate, and weight decay are the hyper-parameter of our model, and we have set them to 0.2, 0.05, and 0.001 respectively in this study. We used softmax cross entropy loss with weight decay for the loss function as shown in [25]. This form of the loss function is the objective for the proposed Bayesian approach. Adam with standard parameters (beta_1 = 0.9, beta_2 = 0.999) were used for the optimizer, and Glorot uniform initializer [26] for the initialization. Transfer learning was not applied in this study because the modality of our dataset is simple – greyscale and small sized images due to cropping – and hence, the architecture of our model is shallow to prevent the possibility of overfitting. Existence of large amount of the augmented training dataset also eliminates the necessity of transfer learning.

### Training set formation

Since the framework was divided into two (LRS and HRS), which required their own convolutional neural network model, each framework had to be trained individually using the appropriate training data. To acquire training materials for LRS, in training set, we used a different data augmentation technique introduced in [17]. According to this method, a large portion of the augmentation is generated through cropping; we set randomly extracted *NT* pixels within the boundary of 18 mm away from each landmark position (landmark-neighboring pixels) to construct a true set, and similarly, *NF* pixels out of the boundary (any pixels that are sufficiently far from the landmark location) to make a false set. We then created a cropped image centered on each pixel as elements of a training batch. For the training dataset of HRS, we take a radius of the true region to be within 0.9 mm and our false region to be within the radius from 2.1 mm to 40 mm [17]. Since the total amount of data was insufficient in the case of the ISBI dataset, we set *NF* = 500 and *NT* = 200 for both LRS and HRS training materials. Therefore, a total of 105,000 (150 raw training image * 700 augmented images-per-raw image) training data were constructed. A total of 38 models (2 screening × 19 landmarks) were trained with 700 training data per training session.

### Statistical analysis

The point to point error of each landmark was measured with the absolute distance and averaged over the entire test set. We defined such value as a landmark error (LE) = $\frac{\sum_{i=1}^n\left\Vert \overset{\rightharpoonup }{m_i}-\overset{\rightharpoonup }{a_i}\right\Vert }{n}\left(\mathrm{mm}\right)$ with $\overset{\rightharpoonup }{m_i}$ and $\overset{\rightharpoonup }{a_i}$ being the manual and estimated landmark position of an image respectively [27]. Standard deviation (SD) among all the test data of a landmark was reported with the error. We also measured the successful detection rates (SDR) which indicate percentages of estimated points within each precision range (z) of 2 mm, 2.5 mm, 3 mm, and 4 mm respectively; SDR = $\frac{\#\left\{i:\left\Vert \overset{\rightharpoonup }{m_i}-\overset{\rightharpoonup }{a_i}\right\Vert <z\right\}}{n}\times 100\ \left(\%\right).$

## Results

### Candidates of landmarks

Candidates of cephalometric landmarks in this study are listed in Table 1. Those which were commonly used for orthodontic diagnosis were chosen to be tested.

**Table 1:** Overall performance of detecting landmarks. The mean landmark error with standard deviation of each landmark, and successful detection rates (SDR) within the 2, 2.5, 3, 4 mm range criteria are listed

	Error (mm)		SDR (%)			
	Mean	SD	2 mm	2.5 mm	3 mm	4 mm
Sella	0.86	1.92	96.67	97.33	98.00	98.00
Nasion	1.28	1.03	81.33	86.00	90.00	96.67
Orbitale	2.11	2.77	77.33	87.33	94.00	96.67
Porion	1.89	1.67	58.00	66.00	72.67	86.67
A-point	2.07	2.53	52.00	62.00	74.00	87.33
B-point	2.08	1.77	79.33	88.67	93.33	96.67
Pogonion	1.17	0.81	82.67	90.67	96.00	100.00
Menton	1.11	2.82	95.33	97.33	98.00	98.67
Gnathion	0.97	0.56	92.00	97.33	98.67	98.67
Gonion	2.39	4.77	63.33	75.33	85.33	92.67
Lower incisal incision	1.35	2.19	84.00	90.67	93.33	96.67
Upper incisal incision	0.90	0.75	93.33	97.33	98.00	99.33
Upper lip	1.32	0.83	96.67	100.00	100.00	100.00
Lower lip	1.28	0.85	97.33	98.67	98.67	99.33
Subnasale	1.22	1.56	84.00	92.00	95.33	96.67
Soft tissue pogonion	2.62	2.07	82.67	92.67	95.33	97.33
Posterior Nasal Spine	1.23	0.91	90.00	94.00	95.33	98.00
Anterior Nasal Spine	1.52	1.56	78.67	87.33	90.67	93.33
Articulare	1.70	1.77	75.33	83.33	86.67	90.67
**Average**	**1.53**	**1.74**	**82.11**	**88.63**	**92.28**	**95.96**

### Landmark annotation results

The developed framework for automatic landmark estimation was compared with the ground truth landmarks of the experienced clinicians. This involves 250 test data given from the ISBI challenge as the previous studies have conducted. A common way to find a good place to stop training is to split a portion of the data set into a validation set to track estimates of generalization performance. However, there is no data for validation in ISBI dataset. Therefore, we found a training stop point through training loss, and selected a point where training loss does not show a significant difference for a certain period. We have attached the plot for the training loss that clarifies the chosen steps in Appendix Fig. 2. In this study, the selected point is on 200,000 steps. One step included 128 image batches.

**Fig. 3:**
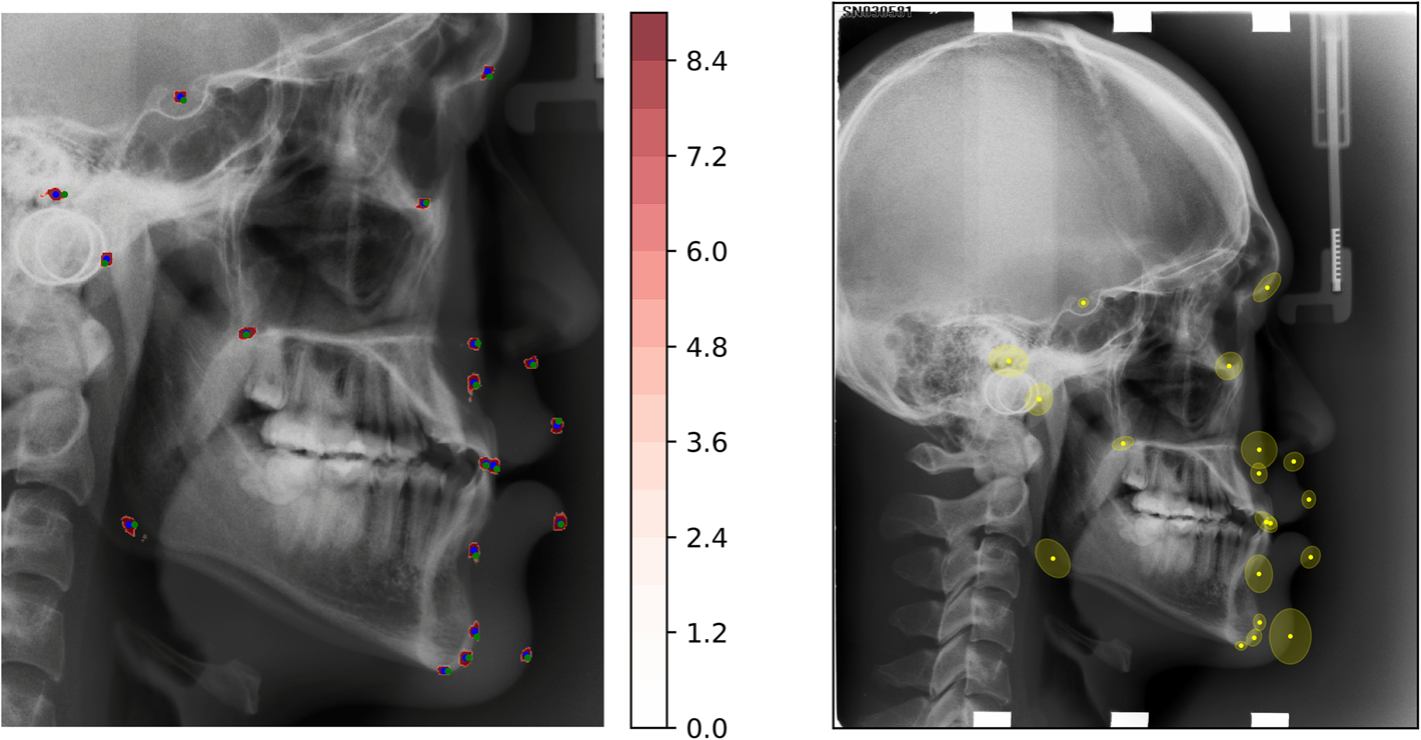
Example of overall outcome. **a** Score plot. Red regions: log-scale score map, Blue dots: estimated positions, Green dots: ground truth. **b** estimated landmarks and confidence regions (95%) in ellipsoid

Figure 3 exemplifies the overall outcome produced from an input cephalogram. The red pixels in Fig. 3a are displayed with a log scale map of previously defined scores. Blue dots represent estimated landmark positions, and ground truth points are plotted with green to be in comparison. In Fig. 3b, ellipsoids around the estimated points denote the corresponding confidence regions (95%) of each landmark.

The manual inter-observer variability between senior and junior doctors produced a LE of 2.02 ± 1.53 on the test set. Whereas our framework has shown its variability with the junior to be 1.79 ± 1.71, and 1.53 ± 1.67 when compared with the ground truth (mean position). Since the produced mean error was less than that of the other clinician-plotted landmarks, the developed framework can obtain its status as a generalized method for determining cephalometric landmarks. More detailed results for the landmark-specific annotations are listed in Table 1. Our framework showed a mean landmark error (LE) of 1.53 ± 1.74 mm and achieved a successful detection rate (SDR) of 82.11, 92.28 and 95.95%, respectively, in the 2, 3, and 4 mm range. In general, ±2 mm errors in cephalometric analysis are clinically acceptable. Errors higher than that can negatively affect orthodontic diagnosis and surgical treatment. The landmark of the largest LE among all was the Soft Tissue Pogonion, with a mean error of 2.62 mm and a SD of 2.07. On the other hand, the landmark which has the smallest LE was Sella, which is very easy to identify its position. Compared to other methods, one of the distinctive features of our framework is the accuracy of the Gonion point. Every method from the ISBI challenge in the year 2015 plots Gonion point the most erroneously, that the smallest error from the ground truth point came out to be more than 4 mm, whereas our framework produced the error of nearly half of the others.

We used four NVIDIA GTX Titan X graphics processing units (GPU) for training and inference. The learning time using one GPU takes about 1.7 h for one landmark, and the total time for learning 19 landmarks and two models (HRS and LRS) is 64.6 (1.7 × 19 × 2) hours. If 4 GPUs are used in parallel, it takes 16.15 h. The inference time using one GPU takes 8 s to perform Bayesian iteration (17 times) for one landmark. Therefore, a total of 512 (8 × 19) seconds is required for 1 image (e.g., 19 landmarks). On the other hand, 38 s is required when using 4 GPUs.

### Classification result

Based on the estimated landmarks, the results were then organized into a form of a confusion matrix to be compared with the ground truth, shown in Table 2. The label that outperforms the other the most was FHI. (84.74%) Out of eight commonly used orthodontic parameters, we achieved four of them to be rated the highest performance among the other methods.

**Table 2:** Confusion matrix of orthodontic parameters for skeletal analysis and their comparison with others’ methods

					Diagonal accuracy		
					Proposed	Lindner et al. (Lindner et al. 2016)	Arik et al. (Arik et al. 2017)
**ANB**	*Pred 1*	*Pred 2*		*Pred 3*	**80.72**	79.90	77.31
*True 1*	**65.75**	10.96		23.29			
*True 2*	23.64	**70.91**		5.45			
*True 3*	4.96	0.83		**94.21**			
**SNB**	*Pred 1*	*Pred 2*		*Pred 3*	**83.13**	78.80	70.11
*True 1*	**73.24**	4.23		22.54			
*True 2*	38.46	**58.97**		2.56			
*True 3*	5.04	0.00		**94.96**			
**SNA**	*Pred 1*	*Pred 2*		*Pred 3*	72.69	**73.81**	66.72
*True 1*	**67.62**	16.19		16.19			
*True 2*	18.89	**80.00**		1.11			
*True 3*	25.93	3.70		**70.37**			
**ODI**	*Pred 1*	*Pred 2*		*Pred 3*	81.53	**81.75**	75.04
*True 1*	**82.14**	6.25		11.61			
*True 2*	33.33	**66.67**		0.00			
*True 3*	15.55	0.91		**85.55**			
**APDI**	*Pred 1*	*Pred 2*		*Pred 3*	84.34	**89.26**	87.18
*True 1*	**82.14**	6.25		11.61			
*True 2*	33.33	**66.67**		0.00			
*True 3*	15.55	0.91		**85.55**			
**FHI**	*Pred 1*	*Pred 2*		*Pred 3*	**84.74**	63.51	69.16
*True 1*	**82.14**	6.25		11.61			
*True 2*	33.33	**66.67**		0.00			
*True 3*	15.55	0.91		**85.55**			
**FMA**	*Pred 1*	*Pred 2*		*Pred 3*	**81.93**	81.92	78.01
*True 1*	**82.14**	6.25		11.61			
*True 2*	33.33	**66.67**		0.00			
*True 3*	15.55	0.91		**85.55**			
**MW**	*Pred 1*	*Pred 3*	*Pred 4*	*Pred 5*	80.32	79.59	**81.31**
*True 1*	**75.00**	1.19	17.86	5.95			
*True 3*	0.00	**89.76**	2.04	8.16			
*True 4*	13.89	0.00	**86.11**	0.00			
*True 5*	15.91	13.64	0.00	**70.45**			

Results are shown as percentage (%)

Abbreviations: *ANB* angle between A-point, nasion and B-point, *SNB* angle between sella, nasion and B-point, *SNA* angle between sella, nasion and A-point, *Overbite depth indicator (ODI)* sum of the angle between the lines from A-point to B-point and from Menton to Gonion, and the angle between the lines from Orbitale to Porion and from PNS to ANS, *Anteroposterior dysplasia indicator (APDI)* sum of the angle between the lines from Orbitale to Porion and from Nasion to Pogonion, the angle between the lines from Nasion to Pogonion and from Subspinale to Supramentale, and the angle between the lines from Orbitale to Porion and from PNS to ANS, *Facial height index (FHI)* ratio of the posterior face height (distance from Sella to Gonion) to the anterior face height (distance from Nasion to Menton), *Frankfurt mandibular angle (FMA)* angle between the lines from sella to nasion and from gonion to gnathion, *Modified Wits Appraisal (MW)* the distance between Lower incisal incision and Upper incisal incision

## Discussion

Cephalometry is used as a very important criterion in the diagnosis and treatment planning of orthodontics. However, the lack of certainty about the definition of cephalometric landmarks causes inter-observer variability due to individual tendencies involved in the measurement. Manual inspection errors due to fatigue can also add to intra-observer variability. Moreover, plotting cephalometric landmark in a manual manner is a time-consuming behavior, and hence should be reduced in their time requirement to be productive. Therefore, it is necessary to establish an automatic framework of orthodontic analysis that can rapidly estimate and analyze accurate and reliable cephalometric landmarks.

We presented the automated framework for detecting cephalometric landmarks which is the first attempt to implement confidence regions (95%) around the estimated positions of landmarks. By providing the confidence regions, the clinician can intuitively gauge the accuracy of the calculated landmark of the system, especially according to its location and size. Landmark detection along with confidence regions will not only inform clinicians of the confidence on the estimated landmarks, but also efficiently reduce time by narrowing areas to be considered for clinicians’ decision making.

A considerable part of the result is that there is a distinct negative correlation between the error and SDR. The correlation is analyzed using the Pearson correlation coefficient [28] and the coefficient between error mean and SDR (2 mm) is − 0.689. It is natural that the larger the error, the lower the SDRs, but exceptionally low SDRs are observed for Porion and A-point. This is due to low tracing accuracy. In fact, the difference between the landmarks indicated by the two experts is 3.31 ± 2.29 and 2.89 ± 2.31 (mm), respectively, which shows a large SD. This means that the tracing position of one or both experts fluctuates greatly. Indeed, there is research showing that Porion and A-point have low reliability of manual tracing [29, 30]. The effect of A-point with low reproducibility can also be seen in Table 2. SNA and SNB have the same components other than A-point or B-point, however, their diagonal accuracy is 72.69 and 83.13, showing a high difference. Labeling errors are inevitable in areas such as landmark detection, where there is no golden standard and where ground truth must be generated through manual labeling. This problem can be mitigated with very large amounts of data or very accurate landmark tracing.

There are landmarks that are difficult to identify: A-point, Articulare, Soft tissue pogonion, Orbitale, and Gonion [11, 20, 23, 31]. These landmarks show higher errors or lower SDRs than other landmarks. We discuss the reasons for the low performance of these landmarks. A-point is a landmark located on the curve of the premaxilla and is often influenced by the head position which makes tracing difficult. It has been discussed in preceding studies that A-point is one of the most common landmarks suffering from errors in identification [29, 32]. Articulare, the intersection of the external dorsal contour of the mandibular condyle and the temporal bone, is an example of the cephalostat affecting the performance. When tracing Articulare, the ear rods of the cephalostat used to fix the head position are also shown on the lateral cephalogram. Those distract the model from the temporal bone. In the case of Soft tissue pogonion, as mentioned in [31], there is a large difference between annotations in test 1 and test 2. In this study, we used both data for testing at once. Therefore, the average performance decreases. In addition, we confirmed the same tendency in the annotation for Orbitale. Due to the limitation of the lateral cephalogram of 2D, when both jaws are not exactly superimposed, Gonion could be marked on the left or right jaw. This could reduce the performance.

There remain several limitations on the framework. Because the model is trained on regional geometrical features only, spatial relationships of landmarks are not considered. This contributes to the aberrant outcome, e.g. B-point plotted inside the mouth. In addition, the proposed framework does not consider contours, so that multiple landmarks lie directly on the edges of the bone and on the skin, especially at the lower jaw. However, by implementing a mechanism which considers spatial feature such as game theoretic framework [11] in the convolution structure, we expect our performance to improve further. Moreover, image preprocessing techniques, such as the Laplacian filter, to make the edges more prominent, can improve the accuracy of landmarks located on the lower jaw.

There are also issues regarding the generalizability and reliability of ground truth. The study considered small data (only 400 patients) but had a wide range of ages (six to 60 years). In addition, the mean intra-observer variability of the two experts was 1.73 and 0.90 mm, respectively, and the manual inter-observer variability between two experts produced a LE of 2.02 ± 1.53 mm on the test set. This is quite large variability, considering that the first precision range is 2 mm. Therefore, there is a high probability that unnecessary bias exists in the trained model, suggesting that there is a limit to clinical applications merely with this dataset. Many high-quality data generated by consensus of several experienced specialists will solve this problem. As an extension of our study, we plan to collaborate with several medical centers to collect patient data from various races and regions in order to build a model that can be applied to everyone.

An automated framework for the detection of cephalometric landmarks using Bayesian BCNN is proposed in this study. It not only has high overall accuracy within 2 mm range prediction but also provides a confidence region (95%) of each landmark. Accuracy rate within 2, 2.5, 3, and 4 mm range are recorded at 82.11, 88.63, 92.28 and 95.96% respectively.

## Conclusions

This study differs from previous studies in that it provides confidence regions for cephalometric landmarks. Our framework may serve as a computer-aided diagnosis tool that improves the accuracy and reliability of decisions by specialists. Improved models that present confidence regions can serve as an efficient and powerful tool for helping unexperienced dentists with cephalometric tracing. In addition, it may be utilized for a training purpose, e.g., training of residents by providing with predicted landmarks as well as their confidence regions.

## Supplementary information

**Additional file 1: METHODS DETAILS. Appendix Fig. 1.** The accuracy plot according to the iteration number. The lowest iteration number that keeps the highest accuracy is 17. **Appendix Fig. 2.** Training curve for cross entropy loss with weight decay [1] in 19 landmarks of HRS (blue dashed line) and LRS (red line) models. One step included 128 image batches.

## Data Availability

The data supporting the results of this study are available in the figshare repository, [https://figshare.com/s/37ec464af8e81ae6ebbf] [23].

## Abbreviations

BCNN: Bayesian Convolutional neural networks
ROI: Region of interest
LE: Landmark error
SDR: Successful detection rate
LBS: Low-resolution screening
HRS: High-resolution screening
CC: Convolutional cluster
FC: Fully connected
SD: Standard deviation
