# Supplementary material for: Automated cephalometric landmark detection with confidence regions using Bayesian convolutional neural networks
Source: BMC Oral Health. 2020 Oct 7;20:270. doi: 10.1186/s12903-020-01256-7 (PMC7541217; doi:10.1186/s12903-020-01256-7)
Supplement: Supplementary file 1 — Additional file 1: METHODS DETAILS. Appendix Fig. 1. The accuracy plot according to the iteration number. The lowest iteration number that keeps the highest accuracy is 17. Appendix Fig. 2. Training curve for cross entropy loss with weight decay [1] in 19 landmarks of HRS (blue dashed line) and LRS (red line) models. One step included 128 image batches. [file 12903_2020_1256_MOESM1_ESM.docx]

**Appendix**

**Automated cephalometric landmark detection with confidence regions using Bayesian Convolutional Neural Networks**

Jeong-Hoon Lee^1*^, Hee-Jin Yu^1*^, Min-ji Kim^3^, Jin-Woo Kim ^2^, Jongeun Choi^1^

^1^School of Mechanical Engineering, Yonsei University, Seoul, Republic of Korea

^2^Department of Oral and Maxillofacial Surgery, School of Medicine, Ewha Womans University, Seoul, Republic of Korea

^3^Department of Orthodontics, School of Medicine, Ewha Womans University, Seoul, Republic of Korea

*Authors contributing equally to this article.

**Corresponding authors**

**Jongeun Choi,** *PhD*

School of Mechanical Engineering, Yonsei University
50 Yonsei Ro, Seodaemun Gu, Seoul 03722, Republic of Korea
Tel: 82-2-2123-2813,  Fax: 82-2-312-2159
*E-mail:* [*jongeunchoi@yonsei.ac.kr*](mailto:jongeunchoi@yonsei.ac.kr)

**Jin-Woo Kim***, DDS, MSD, PhD,FIBSCOMS*

Department of Oral and Maxillofacial Surgery

School of Medicine, Ewha Womans University

Anyangcheon-ro 1071, Yangcheon-gu, Seoul, 158-710, Republic of Korea

Tel: 82-2-2650-2720, Fax: 82-2-2650-2754

*E-mail:* [*jinu600@gmail.com*](mailto:jinu600@gmail.com)*, jwkim84@ewha.ac.kr*

**METHODS DETAILS**

***Bayesian Inference – Uncertainty in Deep Learning***

Uncertainty plays an important role in the process of actual judgment. Even if the algorithm yields the same result, it may be necessary to be made different judgments based on the degree of uncertainty. However, current deep learning models are not interested in uncertainty. Even if a conventional artificial intelligence model trains through precise training data, it does not provide information about uncertainty over the model. Besides, the classification probability of input data derived from the model softmax function cannot be interpreted as the confidence of a model due to the mistranslation between inputs over outputs [1]. Therefore, in this study, we propose a novel algorithm that produces output with high accuracy and some degree of uncertainty from probabilistic measurement methods with low calculation cost and high reliability based on training input samples and models.

There has been a lot of interest in estimating the uncertainty of the model and related research is continuing. Neal, *et al.* proposed an approach to infer the uncertainty by applying the Gaussian distribution to a finite number of neural networks, but the computational complexity was high [2]. An attempt has been made to add a degenerative inference algorithm to the above method [3], but this has also achieved limited success in terms of accuracy. In addition, stochastic variational inference using a sampling technique or batch is applied to degenerative reasoning [4], but the cost for computation is too large to use. Therefore, we decided to use Bayesian inference using the only dropout while maintaining the model [1]. We applied this method to the Convolutional Neural Network to infer the uncertainty. In this method, we can calculate the Bayesian mean and the uncertainty of the model by marginalizing the product of the model posterior probability and the model softmax value for the arbitrary input value against the model parameter ω. In addition to this, the above study shows the physical meaning such that when dropout is applied to a model, learning using the objective function with an *L*2 normalization mechanism is equivalent to minimizing the Kullback-Leibler divergence (*KLDIV*) between the posterior probability of the model through the Gaussian process and the approximated model distribution.

Suppose we have the N images. Let’s define $y_{n}$as an input value of *nth* image, $\hat{y_{n}}$as an output value of the *nth* image from the model, and *E*(.,.) as a cost function according to the model error. In a similar way, if let the [*Wl*] and *bl* be the *lth* kernel weight and bias of total $L$, then cost function with dropout applied is shown as follows

| $L_{dropout}\triangleq\frac{1}{N}\sum_{n=1}^{N} E\left( y_{n},\hat{y_{n}} \right)+\lambda\sum_{l=1}^{L} (\left\Vert\left[ W_{l} \right] \right\Vert_{2}^{2}+\left\Vert b_{l} \right\Vert_{2}^{2})$ | (1) |
| --- | --- |

which give the direction in learning the model, and the posterior probability can be described as

| $p\left( \Theta\vert\left[ X \right], y,x^{*} \right)=\int p\left( \Theta\vert x^{*},\omega\right)p\left( \omega\vert\left[ X \right],\hat{y} \right)d\omega$ | (2) |
| --- | --- |

where [*X*] is a set of learning inputs for posterior probabilities, *y* is a result of learning, and Θ is a set of variables about input data, *x*∗. In this case, if we find a variable set Θ that minimizes the *KLDIV* between the posterior probability $p\left( \Theta| \left[ X \right], y,x^{*} \right)$ and the approximate distribution of any random variables, then the posterior probability can be assumed as the approximate distribution [5]. *KLDIV* of Eq. 2 can be expressed as

| $KL_{DIV}(\left. q_{\Theta}\left( \omega\right) \right\Vert p\left( \Theta\vert\left[ X \right], y,x^{*} \right)=-\int q_{\Theta}\left( \omega\right)\log p\left( \omega\vert\left[ X \right],y \right)d\omega+KL_{DIV}(\left. q_{\Theta}\left( \omega\right) \right\Vert p\left( \omega\right)$ | (3) |
| --- | --- |

Based on the premise that the infinitely wide variable distribution of the neural network converges to the Gaussian distribution, we can define the prior probability of the model as Gaussian distribution.

| $KL_{DIV}=-\sum_{n=1}^{N} \log\frac{e^{f\left( x_{n},\theta\left( \hat{\omega}_{n} \right)_{k} \right)}}{\sum_{j} e^{f\left( x_{n},\theta\left( \hat{\omega}_{n} \right)_{j} \right)}}+\lambda\sum_{l=1}^{L} \left\Vert\left[ W_{l} \right] \right\Vert_{2}^{2}$ | (4) |
| --- | --- |

The integration of *KLDIV* (in Eq. 3) can be approximated by a simple sum of the finite random variable ω by Monte Carlo integration in practical applications. Further, The *KLDIV* between the approximate distribution and the prior probability can be represented by the *L*2 regularization form since the prior probability has the Gaussian distribution. Therefore, if the cost function in Eq. 1 is applied to the model softmax cross entropy, it can be the same form as Eq. 4. This means that for the set of quasi-learning, searching for theta satisfying the approximation of the posterior probability can be regarded as model training. Therefore, we can calculate the mean and uncertainty using the approximate distribution obtained before.

We apply the Bayesian Deep Learning algorithm described in the above to CNN, set the objective function as *KLDIV*, and proceed with model training on the data set. In order to derive the results from the model that has been learned, the input data to be predicted should be inserted and the product of the softmax and the approximate distribution of the model should be marginalized with respect to the random variable ω. This can be expressed as follows

| $p\left( \hat{y}^{*} \vert\left[ X \right],y,x^{*} \right)=\int p\left( y^{*} \vert x^{*},\omega\right)q\left( \omega\right)d\omega$ | (5) |
| --- | --- |

If we consider each ω as a model variable that is arbitrarily changed by drop out each model implementation, the Bayesian mean and uncertainty can be derived as shown in Eq. 6 and Eq. 7, respectively, by the Monte Carlo integration for T times. Here, one execution means one model calculation.

| $\mu_{pred}=\frac{1}{T}\sum_{i=1}^{T} p(y^{*}\vert x^{*},\theta\left( \hat{\omega}_{i} \right))$  $\sigma_{pred}=\frac{1}{T-1}\sqrt{\sum_{i=1}^{T} \left( p\left( y^{*} \vert x^{*},\theta\left( \hat{\omega}_{i} \right) \right)-\mu_{pred} \right)^{2}}$ | (6)  (7) |
| --- | --- |

A model using the above algorithm is called a Bayesian Convolutional Neural Network (BCNN).

***Optimization using Monte Carlo Model Number***

The Bayesian method implies the characteristics of its neural network model to be regarded distinctively at each iterative calculation, due to the dropout applied at the test time which neutralizes the portion of its neurons randomly with a given rate [1]. Since the forward-propagation from the neural network is considered as a conditional probability under a given training data when marginalized, the number of Monte Carlo Model remains a crucial parameter in that the lack of enforcement can lead to the misrepresentation of the probability by the law of large numbers.

However, once it reaches a certain number where more enforcement is regarded as redundant to reproduce the model conditional distribution satisfactorily, additional forward-propagation will only increase the time demand. To find the proper number of model, it is required to achieve knowledge on the aspect of transition regarding the annotation accuracy with respect to the number. Normally, changing a parameter to optimize certain algorithms results in multiple experimental runs. However, because there exists randomness on each iterative calculation, a single experiment with sufficient forward-propagations can be used to reflect the accuracy of each iteration. From the accuracy plot in Appendix Figure 2, it can easily be notice that there is a proportionality with Bayesian iterations until it reaches *17th* calculations. Accuracy gets saturated after then, representing any more forward-propagations is redundant.

Our system is properly optimized, and the following experimental results are based on the optimized setting from above.

Cf) Due to the nature of CNNs, where the computational complexity increases by the square of the kernel size (*O*(*S*2)), it seems more reasonable to reduce the size of a model as much as possible. Therefore, a multi-model algorithm for each individual landmark is proposed, of which constructs an expected region of existence on one side, and examine pixels within this range on the other. Additionally, downsampling of an input image by a factor of 3 can also reduce the calculation cost necessity.

**
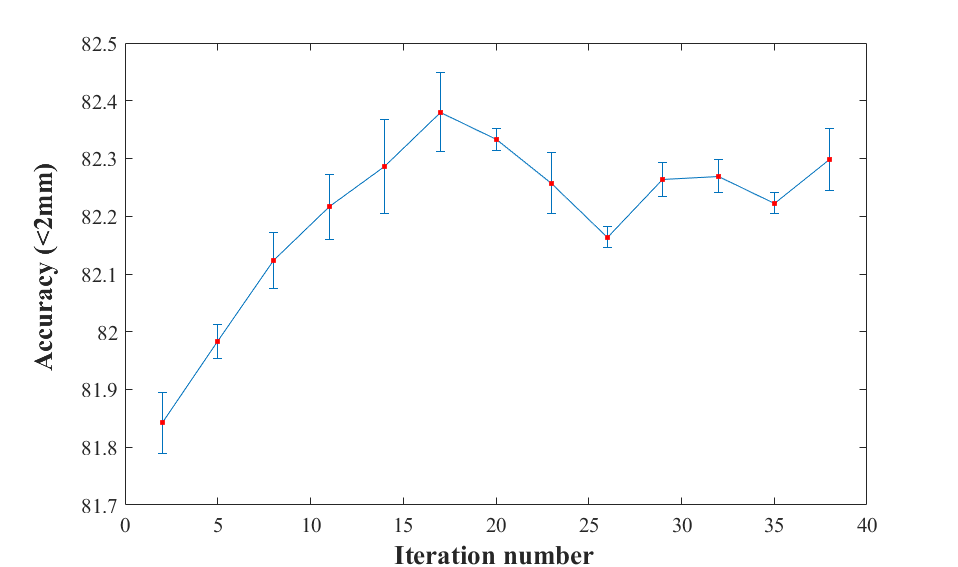
**

**Appendix Figure 1.** The accuracy plot according to the iteration number. The lowest iteration number that keeps the highest accuracy is 17.

**
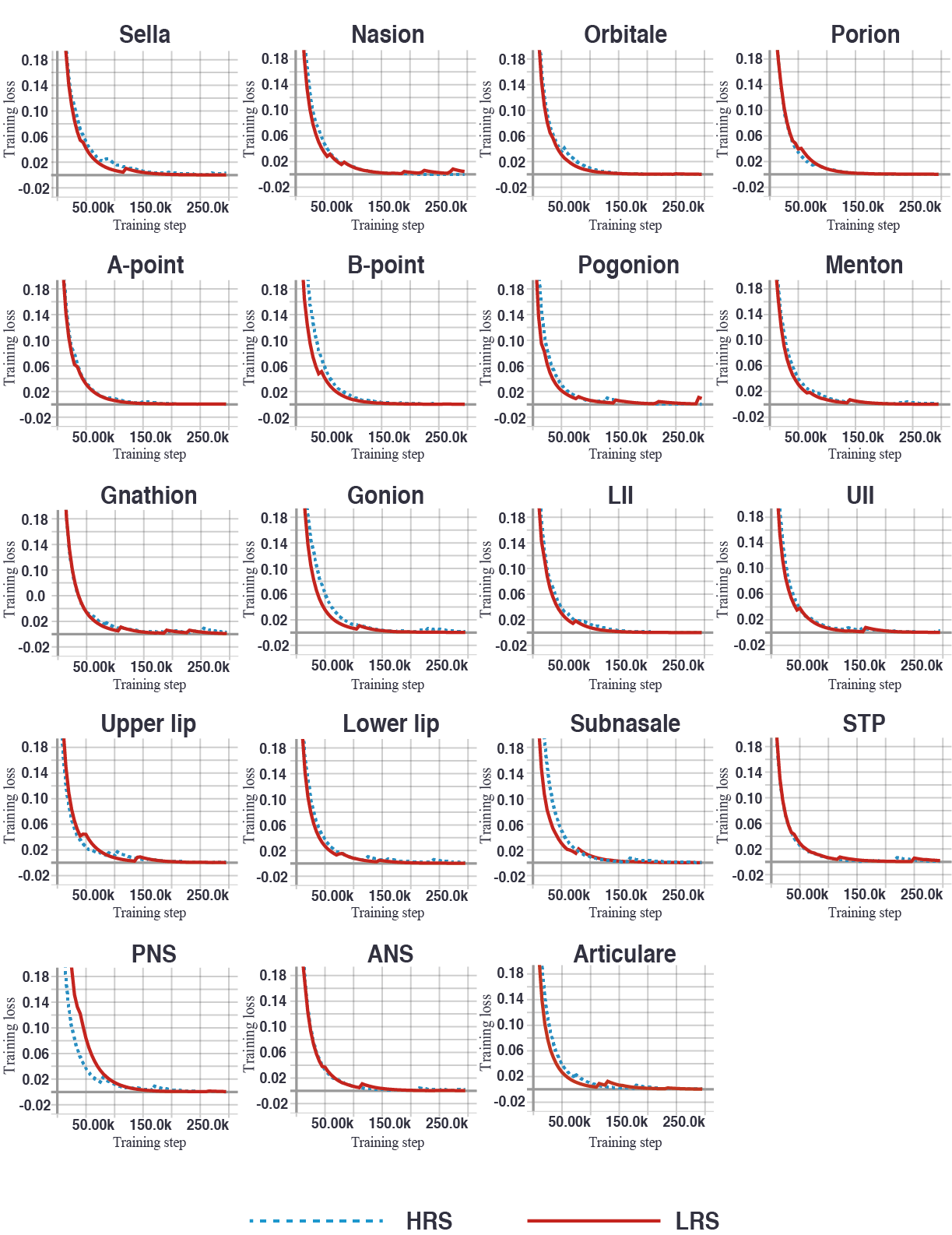
**

**Appendix Figure 2.** Training curve for cross entropy loss with weight decay [1] in 19 landmarks of HRS (blue dashed line) and LRS (red line) models. One step included 128 image batches.

**Abbreviations**

LII: Lower incisal incision, UII: Upper incisal incision, STP: Soft tissue pogonion, PNS: Posterior Nasal Spine, ANS: Anterior Nasal Spine.

**Reference**

1. Gal Y, Ghahramani Z: **Dropout as a bayesian approximation: Representing model uncertainty in deep learning**. *international conference on machine learning* 2016:1050-1059.

2. Neal RM: **Bayesian learning for neural networks**, vol. 118: Springer Science & Business Media; 2012.

3. Hinton G, Van Camp D: **Keeping neural networks simple by minimizing the description length of the weights**. *in Proc of the 6th Ann ACM Conf on Computational Learning Theory* 1993.

4. Hoffman MD, Blei DM, Wang C, Paisley JJTJoMLR: **Stochastic variational inference**. 2013, **14**(1):1303-1347.

5. Kullback S, Leibler RAJTaoms: **On information and sufficiency**. 1951, **22**(1):79-86.
